# Effect of Cadmium Toxicity on Growth, Oxidative Damage, Antioxidant Defense System and Cadmium Accumulation in Two Sorghum Cultivars

**DOI:** 10.3390/plants9111575

**Published:** 2020-11-13

**Authors:** Muhammad Jawad Hassan, Muhammad Ali Raza, Sana Ur Rehman, Muhammad Ansar, Harun Gitari, Imran Khan, Muhammad Wajid, Mukhtar Ahmed, Ghulam Abbas Shah, Yan Peng, Zhou Li

**Affiliations:** 1 Department of Grassland Science, Animal Science and Technology College, Sichuan Agricultural University, Chengdu 611130, China; jawadhassan3146@gmail.com (M.J.H.); Imran.62k@gmail.com (I.K.); pengyanlee@163.com (Y.P.); 2 College of Agronomy, Sichuan Agricultural University, Chengdu 611130, China; Razaali0784@yahoo.com; 3 Department of Agronomy, PMAS-Arid Agriculture University, Rawalpindi 46000, Pakistan; Sana07139@gmail.com (S.U.R.); muhammad.ansar@uaar.edu.pk (M.A.); ahmadmukhtar@uaar.edu.pk (M.A.); shahga@uaar.edu.pk (G.A.S.); 4 Department of Agricultural Science and Technology, School of Agriculture and Enterprise Development, Kenyatta University, Nairobi P. O. Box 43844-00100, Kenya; harun.gitari@ku.ac.ke; 5 College of Plant Science and Technology, Huazhong Agricultural University, Wuhan 430070, China; hafizwajid.agri@gmail.com; 6 Department of Agricultural Research for Northern Sweden, Swedish University of Agricultural Sciences, 90183 Umeå, Sweden

**Keywords:** Cadmium, heavy metal, food security, oxidative damage, antioxidants

## Abstract

Heavy metal stress is a leading environmental issue reducing crop growth and productivity, particularly in arid and semi-arid agro-ecological zones. Cadmium (Cd), a non-redox heavy metal, can indirectly increase the production of reactive oxygen species (ROS), inducing cell death. A pot experiment was conducted to investigate the effects of different concentrations of Cd (0, 5, 25, 50, 100 µM) on physiological and biochemical parameters in two sorghum (*Sorghum bicolor* L.) cultivars: JS-2002 and Chakwal Sorghum. The results showed that various concentrations of Cd significantly increased the Cd uptake in both cultivars; however, the uptake was higher in JS-2002 compared to Chakwal Sorghum in leaf, stem and root. Regardless of the cultivars, there was a higher accumulation of the Cd in roots than in shoots. The Cd stress significantly reduced the growth and increased the electrolyte leakage (EL), hydrogen peroxide (H_2_O_2_) concentration and malondialdehyde (MDA) content in both cultivars, but the Chakwal Sorghum showed more pronounced oxidative damage than the JS-2002, as reflected by higher H_2_O_2_, MDA and EL. Moreover, Cd stress, particularly 50 µM and 100 µM, decreased the activity of different antioxidant enzymes, including superoxide dismutase (SOD), peroxidase (POD), and catalase (CAT). However, the JS-2002 exhibited higher SOD, POD and CAT activities than the Chakwal Sorghum under different Cd-levels. These findings revealed that JS-2002 had a stronger Cd enrichment capacity and also exhibited a better tolerance to Cd stress due to its efficient antioxidant defense system than Chakwal Sorghum. The present study provides the available information about Cd enrichment and tolerance in *S. bicolor*, which is used as an important agricultural crop for livestock feed in arid and semi-arid regions.

## 1. Introduction

Plants are adversely affected by various environmental factors that hinder their growth and development. Heavy metal stress is one of the most critical abiotic factors that has gained enormous attention over the last 30 years [1,2]. A heavy metal is defined as any element exhibiting high density and that exerts its lethal effects even when available in trace amounts. In short, heavy metals belong to a class of metals with an atomic density of more than 4 g cm^−3^ [1]. Among all elements discovered so far, 53 elements have been identified as heavy metals. However, most of them have no beneficial function in plant metabolism. Among them, chromium (Cr), lead (Pb), cadmium (Cd), silver (Ag), cobalt (Co), platinum (Pt), arsenic (As) and nickel (Ni) play the most devastating role in plant physiology [3]. Heavy metals exist in soils naturally in amounts that exert no apparent harm. However, due to an increase in anthropogenic and geological activities in the past few decades, the concentration of such metals in the environment has increased greatly, which is extremely harmful to plant and animal species [3]. Heavy metals restrict plant growth by lowering the performance of different cell components, such as the thylakoid membranes of chloroplast, lipids and proteins [4]. Besides, heavy metals get incorporated into the food chain from plants to animals, thus leading to a potential risk of different disorders in human beings [5].

Among all the heavy metals, Cd has gained extreme importance due to its massive involvement in food chain contamination, as it is easily absorbed by the cells of various plant species [6,7,8]. Cd is readily soluble in water, thus entering the semi-permeable membrane of root cells via an active transport mechanism, following an analogous pathway used for zinc transport [9,10]. Cd ions’ movement across the semi-permeable membrane takes place with the help of metal transporters (ZIP family), which are a special type of transmembrane protein used for the transport of metals across biological membranes [11,12]. Due to advancements in agricultural and industrial sectors, serious attention has been given to issues related to Cd toxicity in the past few decades [13]. Cd presence in excessive amounts (≥1 mM) in the soil is detrimental for plants, resulting in hazardous effects on many physiological processes, including growth inhibition, root browning, chlorophyll degradation and chlorosis of leaves [14,15,16,17,18]. Cd toxicity results from the increased concentration of Cd ions in both roots and shoots, thus decreasing the crop biomass [19,20,21,22]. Agricultural crops absorb Cd via their roots from water present in the soil, so that its uptake correlates with the total Cd available in soil solution [23,24,25,26]. Heavy metal accumulation, especially Cd, has been thoroughly investigated, but the exact mechanism involved in Cd stress injury to plants is still not clearly understood.

Cd toxicity is the leading cause of oxidative damage, resulting in the reduced growth of plants by inducing changes in membrane permeability and the later production of reactive oxygen species at the organelle level. The different types of reactive oxygen species (ROS) formed due to Cd stress include hydrogen peroxides (H_2_O_2_), hydroxyl radical (OH^−^) and superoxide anion (O^2−^), and these are responsible for membrane damage [27,28]. These ROS are the primary causes of membranous proteins and lipids oxidation, which are associated with cell death [29,30]. However, plants have a natural defense system consisting of enzymatic and non-enzymatic antioxidants to protect them against oxidative damage. Enzymatic antioxidants include superoxide dismutase (SOD), peroxidase (POD), catalase (CAT) and ascorbate peroxidase (APX), while non-enzymatic antioxidants consist of glutathione (GSH), ascorbate (ASA), carotenoids and α-tocopherol, which are an effective defensive mechanism to safeguard plants against stress conditions [27,31]. At the cellular level, the first step in the antioxidant defense mechanism is the production of superoxide dismutase for scavenging O^2−^. The CAT and APX play an important role in H_2_O_2_ quenching [32,33,34]. Many studies have been published concerning the defensive role of antioxidants against Cd stress in plants [35,36].

Sorghum (*Sorghum bicolor*) stands at the fifth position among the economically important crops worldwide, while its number is third in the USA. It is a multipurpose crop that can be used not only for food, fodder, and feed, but also as an important ingredient in the beverage industry and in biofuels [35]. Due to its high climatic adaptability, greater fodder yield potential, better quality, palatability, digestibility and bioactive compounds content, sorghum has been widely cultivated in south-Asia, Africa and Central America as an excellent animal feed [35]. It is also a rich source of various phenolic compounds (luteolinidin, apigeninidin, naringenin, etc.) and essential vitamins such as vitamin E [36]. Furthermore, sorghum is tolerant to various abiotic stresses, including high temperature, drought, and heavy metal toxicity. Studies have shown that sorghum plants accumulate large quantities of heavy metals in their roots and shoots with higher biomass production compared to other summer crops [37,38]. The ‘JS-2002’ and ‘Chakwal Sorghum’ are two important sorghum cultivars that are widely used as forages to feed livestock under the arid and semiarid regions of Pakistan. The objectives of this study were therefore to evaluate the impact of Cd toxicity on physiological and biochemical parameters, such as growth, Cd accumulation, oxidative damage and antioxidative enzyme activities, in two sorghum cultivars during the stress period. These findings will provide available information about Cd enrichment and tolerance in sorghum, which is used as an important agricultural crop for livestock feed in arid and semi-arid regions. The results will also help to estimate forage safety when the sorghum is cultivated in Cd contaminated soil.

## 2. Materials and Methods

### 2.1. Planting Material and Treatments

The present experiment was conducted during the summer season of 2017 in a research area of the Agronomy Department Pir Mehr Ali Shah Arid Agriculture University, Rawalpindi, Pakistan. The study area was located at 33.649° N latitude and 73.082° E longitude. Seeds of two sorghum cultivars, viz. JS-2002 and Chakwal Sorghum, were obtained from National Agriculture Research Centre Islamabad. Before sowing, seeds (50 g for each cultivar) were surface sterilized with 75% ethanol for 10 min and thoroughly washed with distilled water. Both JS-2002 and Chakwal Sorghum are diploids. Seeds were sown in plastic pots (18 cm diameter and 22 cm depth) containing 5 kg of sandy loam soil that was collected from the research fields of the Department of Soil Sciences, PMAS Arid Agriculture University Rawalpindi, Pakistan. Eight seeds were sown in each pot and then thinned to three at the four-leaf stage. The pH of the soil was 7.3 with total Cd (0.21 mg/Kg), and pots were arranged under a completely randomized design with three replicates. Plants were watered daily to maintain field capacity and avoid drought stress. Four different Cd concentrations (5, 25, 50, and 100 μM) were fertigated in the form of CdCl_2_ before sowing, while a control (without Cd) was kept for comparison. A 400 mL per kg of Cd solution was irrigated evenly in each plastic pot. At 55 days after sowing (DAS), fully expanded and mature leaves were collected for the measurement of morphological parameters, oxidative damage associated with the reactive oxygen species and activities of different antioxidant enzymes. Leaf samples were dissected and immediately immersed in liquid nitrogen then stored at −80 °C in the freezer for further analysis. Similarly, leaf, stem and root samples were collected at 55 DAS for the measurement of Cd accumulation.

### 2.2. Measurement of Growth Parameters

The height and the leaf number of each sorghum plant were measured at the 55th day after sowing to obtain a clear view of Cd toxicity.

### 2.3. Estimation of Cd Accumulation

Fresh root, stem and leaf samples at the heading stage were thoroughly washed with deionized water. Later, samples were dried in an oven for 24 h at 70 °C, then converted to powder form and placed in a muffle furnace for 4 h at 550 °C for dry-ashing. After this, the obtained ash residues were added with 1M nitric acid to make a standard volume. The amount of Cd accumulated was measured by using a flame atomic absorption spectrophotometer (Unicam, 929 AAS).

### 2.4. Determination of Cell Membrane Stability and Oxidative Damage

For each treatment, three plants were taken to measure the electrolyte leakage (EL) of leaves [39]. The leaf samples (1 g) were washed with distilled water to clean the contamination from the surface. These were kept in closed vials containing 10 mL of distilled water, incubated in a rotary shaker at 25 °C for 24 h, and the electrical conductivity (EC) of the solution (*L*1) was estimated. Later, samples were autoclaved for 20 min at 120 °C, and final electrical conductivity (*L*2) was achieved after equilibration at 25 °C. The EL (%) was measured as shown in Equation (1).

(1) $$\mathrm{EL}\;\left(\%\right)=\;\frac{L1}{L2}\;\times 100$$

Malondialdehyde (MDA), as the final product of lipid peroxidation, was estimated following the procedure of [40], with minor changes. The reaction mixture comprised 500 µL of enzyme extract, 0.65% (w/v) thiobarbituric acid (TBA) in 20% trichloroacetic acid (TCA), which was heated for thirty minutes, then quickly cooled to stop the reaction. After this, the reaction mixture was centrifuged at 10,000× *g* for ten minutes. The absorbance value of the reaction mixture was recorded spectrophotometrically at 532 nm, while the non-specific absorption value at a wavelength of 600 nm was deducted from absorbance value gradually.

MDA content was calculated using Equation (2).

(2) $$\mathrm{MDA}\;\mathrm{content}=C\times \;\frac{V}{W}$$

where *W* represents ‘sample fresh weight’, *V* represents ‘extract volume’, while *C* is the ‘variation in non-specific absorption between two wavelengths’. It is given as mmol g^−1^ FW.

Hydrogen peroxide (H_2_O_2_) content was estimated by following the method described by [41]. Exactly 0.3 g of fresh leaf samples were taken and ground mechanically using 4 mL ice-cold acetone (CH_3_COCH_3_). The homogenate was centrifuged at 12,000× *g* for 20 min at 4 °C. Then, the supernatant was collected and mixed in 5% (w/v) ammonium hydroxide and titanyl sulphate solution. The mixture was centrifuged again at 3000× *g* for 10 min at 4 °C. After this, the obtained supernatant was thoroughly mixed in 2 M sulphuric acid, and the absorbance was recorded at 415 nm. H_2_O_2_ content was measured using a standard curve.

### 2.5. Estimation of Antioxidant Enzyme Activity

Fresh leaf samples (0.4 g) were homogenized with 2 mL of ice-cooled 100 mM Na_2_PO_4_ buffer (pH 7.8) comprising 1.0% polyvinylpyrrolidone (PVPP) and 0.1 mM EDTA. The mixture was later centrifuged at 12,000× *g* for 20 min at 4 °C. After centrifugation, the supernatant was collected and utilized for various antioxidant enzymes analysis. The activity of SOD was estimated spectrophotometrically at 560 nm by measuring the potential of an enzyme to inhibit the photochemical reduction of nitroblue tetrazolium (NBT) by O^−2^ radicals liberated by light-induced chemical reactions [42]. The POD was determined spectrophotometrically by recording the fluctuation in absorbance value at 470 nm resulting from guaiacol oxidation [43]. The CAT activity was measured by observing the decomposition rate of hydrogen peroxide at 240 nm [43].

### 2.6. Statistical Analysis

Statistical analysis was carried out using statistix 8.1 (version 8.1. Statistix, Tallahassee, FL, USA). Significant differences among all treatments were measured by using ANOVA (one way) in combination with LSD test. The significance of differences was assessed at the 5% probability level (*p* < 0.05).

## 3. Results

### 3.1. Effects of Exogenous Cadmium on Growth Parameters

**Figure 1:**
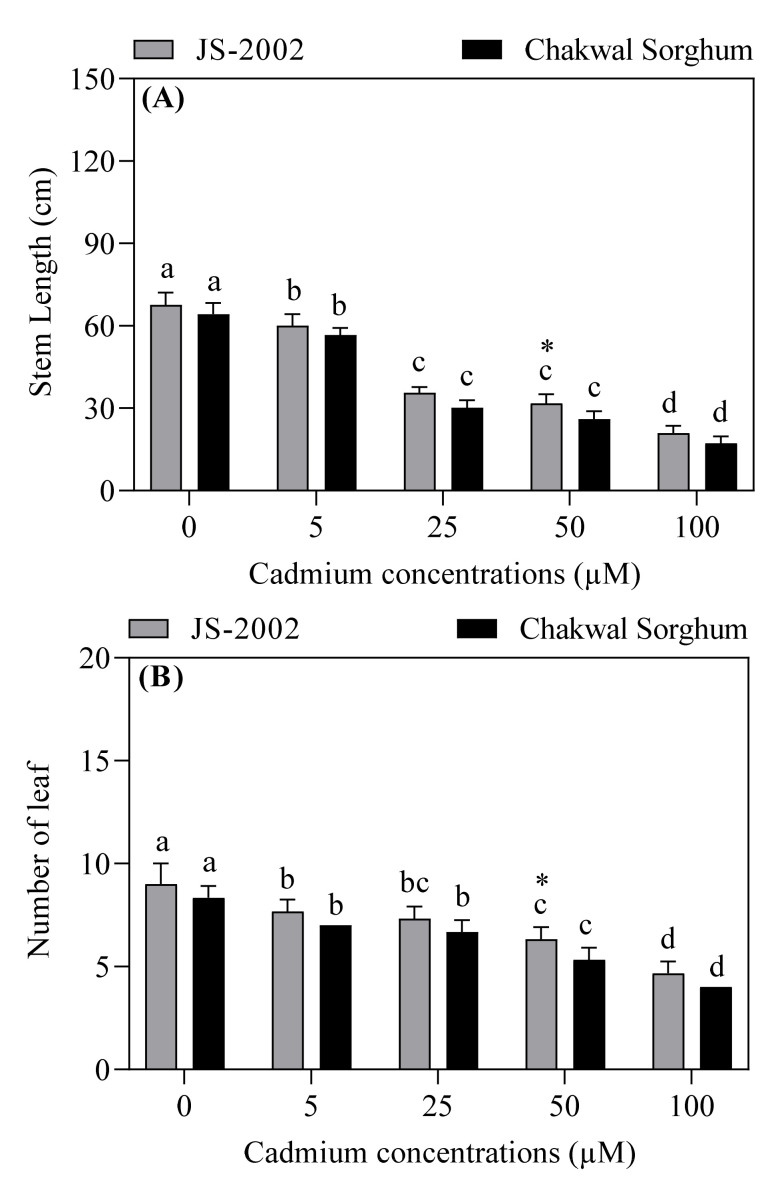
Effect of cadmium toxicity on (**A**) stem length and (**B**) number of leaves in two sorghum cultivars. Values are mean ± standard error (*n* = 5). Different letters in vertical column show significant differences for a cultivar under different cadmium concentrations, whereas “*” shows a significant difference between two sorghum cultivars under a particular cadmium concentration. Comparison of mean was confirmed by LSD at *p* < 0.05.

Different concentrations of Cd significantly affected the plant height and leaf number in both sorghum cultivars (Figure 1). Among the treatments, the highest plant heights (68 or 64 cm) and leaf numbers (9 or 8) were reported in two controls, whereas the respective lowest plant heights (21 or 17 cm) and leaf numbers (5 or 4) were recorded in JS-2002 or Chakwal Sorghum under 100 µM Cd treatment. The results showed that JS-2002 exhibited significantly higher plant height and number of leaves, particularly under the 50 µM treatment, than Chakwal Sorghum, as shown in Figure 1. When compared with the respective control, Cd stress reduced the plant height of the JS-2002 cultivar by 11%, 47%, 53% or 69% in treatments receiving Cd concentrations of 5, 25, 50 and 100 μM, respectively, whereas the respective reductions in the height of the Chakwal Sorghum cultivar were 12%, 53%, 59% and 73%. Moreover, the interactive effect of Cd and plant growth was antagonistic, since plant height and leaf number reduced greatly with a gradual increase in Cd concentration (Figure 1).

### 3.2. Effects of Exogenous Cadmium on Cadmium Accumulation

**Figure 2:**
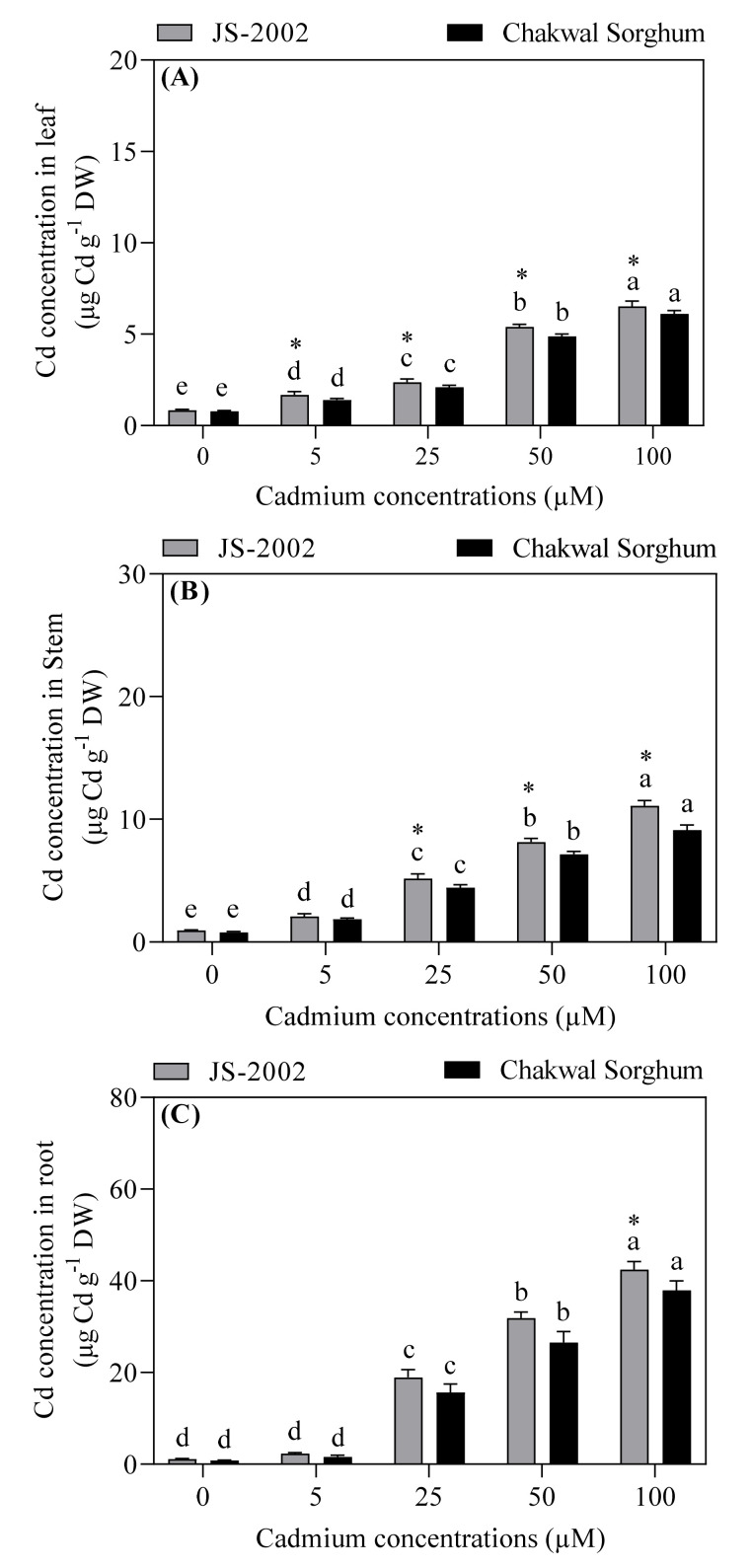
Effect of cadmium toxicity on cadmium accumulation in (**A**) leaf, (**B**) stem, and (**C**) root of two sorghum cultivars. Values are mean ± standard error (*n* = 5). Different letters in the vertical column show significant differences for a cultivar under different cadmium concentrations, whereas “*” shows a significant difference between two sorghum cultivars under a particular cadmium concentration. Comparison of mean was confirmed by LSD at *p* < 0.05.

The highest (42.44 or 37.9 µg Cd g^−1^ DW) Cd accumulation was found in roots, while the lowest value (6.83 or 6.31 µg Cd g^−1^ DW) was observed in leaves of JS-2002 or Chakwal Sorghum (Figure 2). In contrast to the control, different Cd stress levels significantly increased Cd accumulation in various plant organs (leaf, stem and root) of both cultivars, however such an increment was more pronounced when Cd was applied at 50 or 100 µM, respectively. Moreover, JS-2002 showed a significant difference in Cd accumulation compared to Chakwal Sorghum in all plant organs under different levels of Cd toxicity (Figure 2).

### 3.3. Effects of Exogenous Cadmium on Cell Membrane Stability and Oxidative Damage

**Figure 3:**
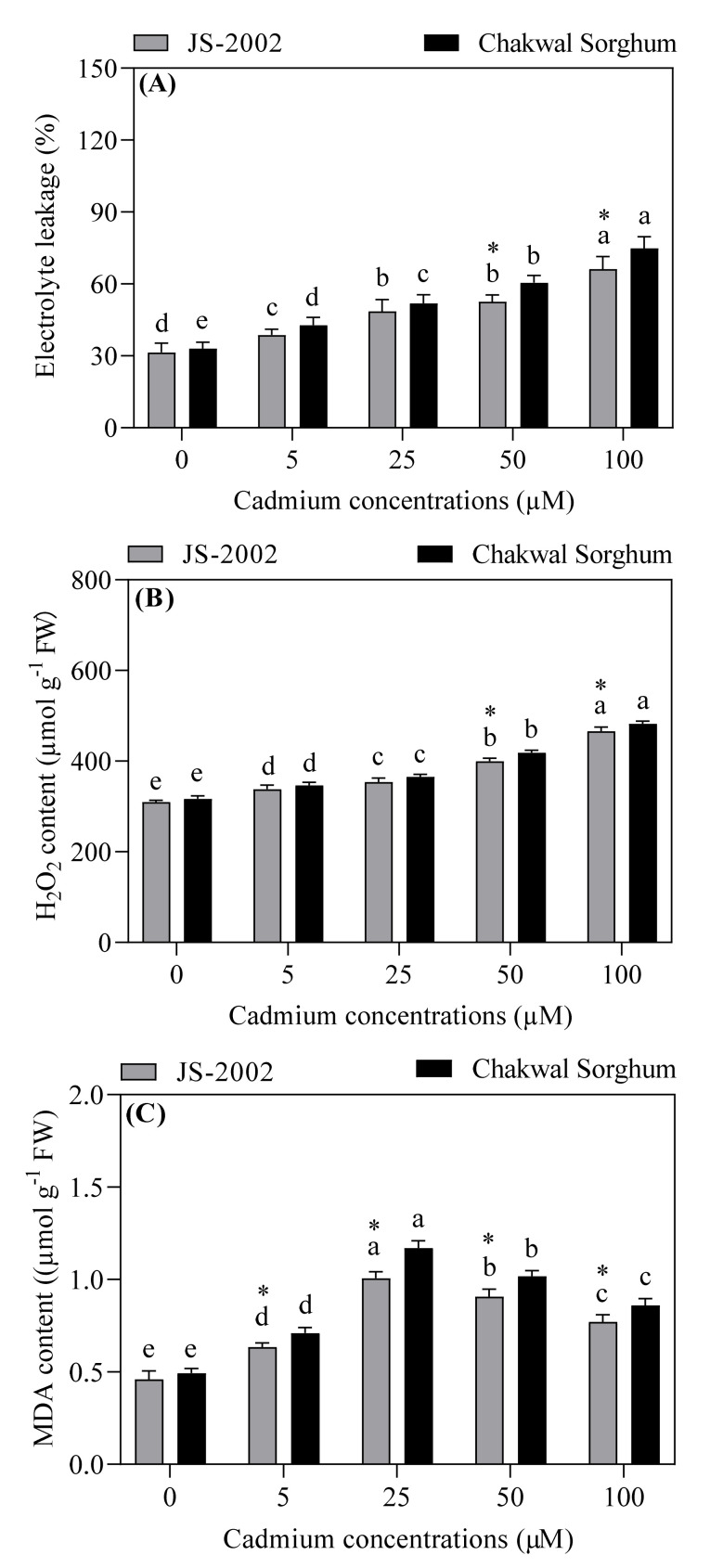
Effect of cadmium toxicity on (**A**) electrolyte leakage (EL), and (**B**) hydrogen peroxide (H_2_O_2_) or (**C**) malondialdehyde (MDA) content in two sorghum cultivars. Values are mean ± standard error (*n* = 5). Different letters in vertical column show significant differences for a cultivar under different cadmium concentrations, whereas “*” shows a significant difference between two sorghum cultivars under a particular cadmium concentration. Comparison of mean was confirmed by LSD at *p* < 0.05.

The Cd application significantly enhanced membrane damage, which intensified with the increase in Cd dosage (Figure 3A). The JS-2002 exhibited considerably lower EL as compared to the Chakwal Sorghum under 50 and 100 µM Cd levels, while no significant difference was noticed in all other treatments, as shown in (Figure 3A). The Cd toxicity induced the massive production of ROS resulting in oxidative stress. The results showed that Cd application significantly elevated the H_2_O_2_ content in the leaves of two sorghum cultivars. However, the JS-2002 showed a significantly lower H_2_O_2_ concentration as compared with Chakwal Sorghum (50 and 100 µM Cd levels). Maximum values of 466 µmol g^−1^ FW and 482 µmol g^−1^ FW were observed in the 100 µM treatment, while the corresponding lowest values of 309 and 317 µmol g^−1^ FW were recorded in the control of JS-2002 and Chakwal Sorghum (Figure 3B). Based on the results of MDA contents in both cultivars grown under 5, 25, 50, or 100 µM Cd stress, a 27%, 54%, 47%, or 39% increase in the JS-2002 and a 31%, 58%, 51%, or 43% increase in the Chakwal Sorghum was found in contrast to their respective control treatment (Figure 3C). However, JS-2002 exhibited significantly lower MDA content as compared to Chakwal Sorghum under all Cd treatments.

### 3.4. Effect of Exogenous Cadmium on Antioxidant Enzymes Activity

**Figure 4:**
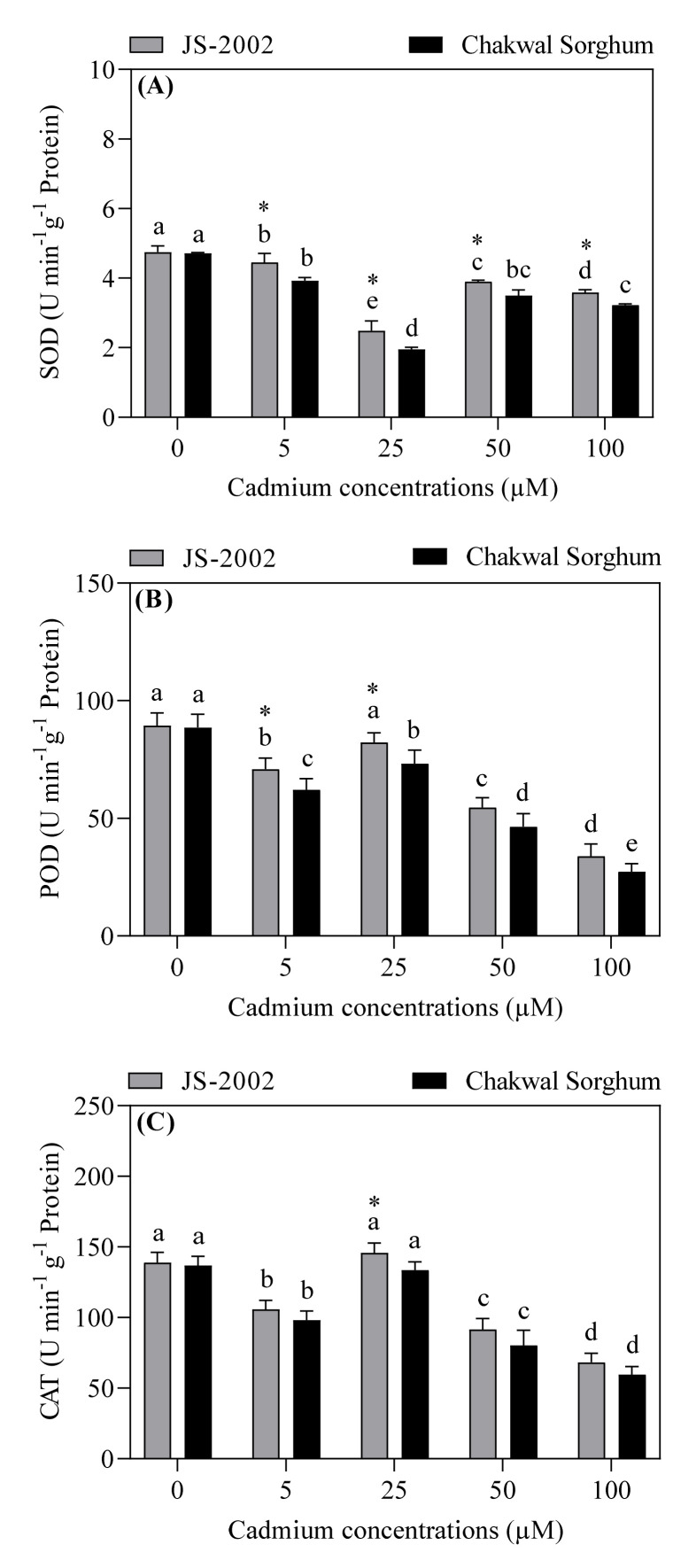
Effect of cadmium toxicity on (**A**) superoxide dismutase (SOD), (**B**) peroxidase (POD), and (**C**) catalase (CAT) activities in leaves of two sorghum cultivars. Values are mean ± standard error (*n* = 5). Different letters in vertical column show significant differences for a cultivar under different cadmium concentrations, whereas “*” shows a significant difference between two sorghum cultivars under a particular cadmium concentration. Comparison of mean was confirmed by LSD at *p* < 0.05.

Significant differences were observed in the antioxidant enzyme activities in the leaves of two sorghum cultivars grown under different levels of Cd stress (Figure 4). The JS-2002 exhibited significantly higher SOD activity under all Cd treatments, while POD and CAT showed significant difference under the 5 or 25 µM treatments when compared to Chakwal Sorghum. Except for controls, the maximum values of POD and CAT activities were recorded in the 25 µM Cd treatments in both sorghum cultivars, however for SOD activity, the maximum values were observed in the 5 µM Cd treatments (Figure 4A–C). The SOD activity increased by 12%, 22%, 28% or 9% in JS-2002 compared to that in the Chakwal Sorghum under 5, 25, 50 or 100 µM Cd stress, respectively (Figure 4A). Moreover, the JS-2002 showed a 12%, 11%, 15%, or 19% increase in POD and a 7%, 8%, 12% or 13% increase in CAT in contrast to the Chakwal Sorghum under 5, 25, 50, or 100 µM Cd stress, respectively (Figure 4B,C).

## 4. Discussion

Cd is a heavy metal with no apparent beneficial role in plant metabolism [14]. Stunting is the most usual and visible response when plants are exposed to Cd stress, which could be correlated with the Cd ion toxicity in plant metabolism, or a Cd induced interference in the uptake of several bio-elements that are essential for growth and development [19]. It has been reported that phosphorus deficiency was the main reason for stunted growth in plants under Cd stress, because Cd and phosphorus form insoluble complexes [19]. A lack or shortage of photosynthetic machinery (leaf) results in the inhibition of organic metabolites, thus leading toward stunted growth under heavy metal stress [44]. Our results showed that Cd application reduced the growth of two sorghum cultivars, JS-2002 and Chakwal Sorghum, as evident from a significant decrease in plant height and leaf number, with pronounced hazardous effects at higher levels of stress. Our results are in line with the study of Daud et al. [45] who observed a linear decrease in plant height and leaf width in cotton cultivars under heavy metal stress.

Plant species vary significantly in their potential to absorb Cd from the soil and transport Cd towards different organs [46]. The amount of Cd absorbed by the roots and Cd translocated towards the shoot depend on its bonding with the extracellular matrix, root efflux, complexion within cells and transport efficiency [47,48]. In the present study, it was observed that the roots accumulated more Cd than the stems or leaves in JS-2002 and Chakwal Sorghum. This possibly could be connected to the fact that the root is the first organ coming into contact with the Cd ions in soil. In addition, the JS-2002 accumulated a higher concentration of Cd in the root, stem and leaves compared to Chakwal Sorghum under Cd stress, which is in accordance with the findings of Wang et al. [26]. At the root level, the first resistance against Cd stress may be provided by the cell wall and extracellular carbohydrates that play an important role in reducing Cd uptake and transport [49]. The Cd accumulation in the roots often hinders the uptake and translocation of other bio-elements, which aggravates Cd toxicity to plants [50].

Abiotic stresses, such as heavy metals, drought, high temperature and salt stress, initially disrupt cell membrane integrity, resulting in increased membrane damage [51,52,53,54]. EL is an imperative index in cell stress physiology and is used to evaluate the leakage of cell components. The results from this study showed that Cd toxicity to both sorghum cultivars enhanced the ROS production, leading to a noticeable increase in membrane damage. Our results are similar to the previous findings in pea and barley [46,55], respectively. Significant increases in membrane damage of Chakwal Sorghum might be connected to the strong imbalance between ROS production and the activity of antioxidative enzymes for ROS scavenging. Cd is considered a non- redox heavy metal that lacks the potential to take part in Fenton-type reactions. Much clear evidence has shown that Cd can indirectly increase the production of ROS through disturbance of electron transport, which is one of the main events taking place in photosystem II [56]. In our study, the application of different Cd levels significantly elevated the H_2_O_2_ concentration in both sorghum cultivars compared to control, however the MDA content first increased and then decreased with the gradual increase in Cd concentration (Figure 3B,C). Our results regarding the increase in MDA and H_2_O_2_ concentration were in accordance with a previous study on wheat [28] and rice in response to Cd stress [57]. In addition, the degree of oxidative damage was lower in JS-2002 in contrast to that in Chakwal Sorghum under all Cd treatments (Figure 3), which could be associated with the more efficient ROS scavenging defense system in JS-2002.

Plants are provided with a natural defense system consisting of enzymatic and non-enzymatic antioxidants to protect them against the oxidative damage induced by various environmental stresses. SOD plays the most important role in the antioxidant defense mechanism, because it is the most effective enzyme in stress resistance, involved in the dismutation of O^2−^ into H_2_O_2_ and molecular oxygen in plants under stress conditions [58]. In the present study, it was observed that the SOD activity in both cultivars was initially decreased by Cd application. However, the JS-2002 exhibited significantly higher SOD activity than the Chakwal Sorghum under all Cd concentrations. Interestingly, the SOD activity firstly declined and then increased in response to 5, 25, 50 and 100 μM Cd concentrations, which is in line with the findings of Liu et al. [59] in Sorghum. The possible reason could be associated with the increased production of O^2−^ radicals, leading to the activation of existing enzyme stock [58]. In addition, a large amount of H_2_O_2_ accumulation is extremely harmful for cell metabolism. The CAT and POD enzymes are responsible for the conversion of H_2_O_2_ to water and oxygen by dissociation of H_2_O_2_, and thus play necessary roles in providing tolerance to unfavorable conditions in plants [53,60,61]. Our findings showed that the JS-2002 maintained higher POD and CAT activities than the Chakwal Sorghum under Cd toxicity (25 µM), indicating the better antioxidant capacity of JS-2002. Interestingly, the CAT and POD activities increased significantly in the leaves under 25 µM Cd stress in both cultivars, as compared to other Cd stress concentration (5, 50 and 100 μM). Considerable increases in POD and CAT activities could be induced by excessive production of H_2_O_2_ (a by-product of superoxide dismutation by SOD) under 25 µM Cd stress. However, these responses were limited due to the severe oxidative damage under higher Cd concentrations (50 and 100 µM) in sorghums. Previous studies on other plant species have also shown that changes in antioxidant enzyme activities were associated with the severity of Cd stress [27,57,58].

## 5. Conclusions

In the present study, different Cd doses were used to investigate the effects on growth, Cd accumulation, oxidative damage, and antioxidative defense system in two sorghum cultivars. The results found out that the majority of Cd was enriched in the roots, and the JS-2002 had a greater Cd enrichment capacity than the Chakwal Sorghum in all plant organs (leaf, stem, and root). In addition, the JS-2002 also showed a better tolerance to Cd stress in comparison with the Chakwal Sorghum, which could be associated with higher antioxidative enzyme activities to cope with the Cd-induced oxidative damage. The current study therefore provides a better understanding of the concentration-dependent role of Cd in sorghum plants; however, intensive work is still required to explain the interaction of Cd with various physiological and genetic functions in sorghum or other plant species.
